# Radiation therapy dose and androgen deprivation therapy in localized prostate cancer: a meta-regression of 5-year outcomes in phase III randomized controlled trials

**DOI:** 10.1038/s41391-021-00432-2

**Published:** 2021-08-16

**Authors:** Tommy Jiang, Daniela Markovic, Jay Patel, Jesus E. Juarez, Ting Martin Ma, David Shabsovich, Nicholas G. Nickols, Robert E. Reiter, David Elashoff, Matthew B. Rettig, Nicholas G. Zaorsky, Daniel E. Spratt, Amar U. Kishan

**Affiliations:** 1 Department of Radiation Oncology, David Geffen School of Medicine, University of California, Los Angeles, CA USA; 2 Department of Urology, David Geffen School of Medicine, University of California, Los Angeles, CA USA; 3 Department of Medicine, Statistics Core, University of California, Los Angeles, CA USA; 4 Division of Hematology and Oncology, David Geffen School of Medicine, University of California, Los Angeles, CA USA; 5 Division of Hematology and Oncology, VA Greater Los Angeles Healthcare System, Los Angeles, CA USA; 6 Department of Radiation Oncology, Penn State Cancer Institute, Hershey, PA USA; 7 Department of Radiation Oncology, University of Michigan, Ann Arbor, MI USA

**Keywords:** Outcomes research, Cancer therapy, Prostate cancer

## Abstract

**Background:**

While multiple randomized trials have evaluated the benefit of radiation therapy (RT) dose escalation and the use and prolongation of androgen deprivation therapy (ADT) in the treatment of prostate cancer, few studies have evaluated the relative benefit of either form of treatment intensification with each other. Many trials have included treatment strategies that incorporate either high or low dose RT, or short-term or long-term ADT (STADT or LTADT), in one or more trial arms. We sought to compare different forms of treatment intensification of RT in the context of localized prostate cancer.

**Methods:**

Using preferred reporting items for systemic reviews and meta-analyses (PRISMA) guidelines, we collected over 40 phases III clinical trials comparing different forms of RT for localized prostate cancer. We performed a meta-regression of 40 individual trials with 21,429 total patients to allow a comparison of the rates and cumulative proportions of 5-year overall survival (OS), prostate cancer-specific mortality (PCSM), and distant metastasis (DM) for each treatment arm of every trial.

**Results:**

Dose-escalation either in the absence or presence of STADT failed to significantly improve any 5-year outcome. In contrast, adding LTADT to low dose RT significantly improved 5-year PCSM (Odds ratio [OR] 0.34, 95% confidence interval [CI] 0.22–0.54, *p* < 0.001) and DM (OR 0.35, 95% CI 0.20–0.63. *p* < 0.001) over low dose RT alone. Adding STADT also significantly improved 5-year PCSM over low dose RT alone (OR 0.55, 95% CI 0.41–0.75, *p* < 0.001).

**Conclusion:**

While limited by between-study heterogeneity and a lack of individual patient data, this meta-analysis suggests that adding ADT, versus increasing RT dose alone, offers a more consistent improvement in clinical endpoints.

## Brief communication

Radiation therapy (RT) dose-escalation for prostate cancer (PCa) has been shown to improve biochemical control in multiple randomized trials but has not been shown to improve clinical outcomes [1]. Adding or prolonging the use of concomitant androgen deprivation therapy (ADT), however, has been shown to improve clinical outcomes in multiple randomized trials [2]. These two methods of treatment intensification have generally been studied independently. To explore the relative benefits of RT dose intensification and adding or prolonging the use of concomitant ADT, we conducted a meta-regression of 40 phases III randomized trials.

The literature review was performed in accordance with the preferred reporting items for systematic reviews and meta-analyses (PRISMA) guidelines (Supplemental Fig. 1) [3]. Each arm of the trials was categorized into the following treatment strategies: low dose RT alone (*n* = 5401 patients from 15 trials), high dose RT alone (*n* = 5009 patients from 11 trials), low dose RT + short-term ADT (STADT, *n* = 6482 from 11 trials), low dose RT + long-term ADT (LTADT, *n* = 2760 patients from 6 trials), and high dose RT + STADT (*n* = 1777 patients from 5 trials). Doses higher than 74 Gy were considered “high dose” (presuming an α/β of 3.0 to convert hypofractionated schedules), while STADT was defined as ≤8 months and LTADT was defined as ≥18 months (Table 1). Extracted data included time period of study (defined as the chronological midpoint of the enrollment period), number of patients, median follow-up time, radiation dosage, length of ADT treatment, percentage of patients with high-risk PCa as defined by the national comprehensive cancer network (NCCN), 5-year overall survival (OS), the 5-year incidence of prostate cancer-specific mortality (PCSM), and 5-year incidence of distant metastasis (DM). OS, PCSM, and DM data were extracted directly from the text, tables, or survival curves using the DigitizeIt Version 2.5 software [4]. Crude incidences were not used for any endpoint.

**Table 1:** Adjusted meta-regression comparing 5-year cumulative proportions of overall survival, prostate cancer-specific mortality, and distant metastasis.

	5-year OS		5-year PCSM		5-year DM	
	OR (95% CI)	*p*-value	OR (95% CI)	*p*-value	OR (95% CI)	*p*-value
*Comparisons with Low Dose RT*						
Low Dose RT + STADT vs. Low Dose RT	1.11 (0.91–1.35)	0.296	0.55 (0.41–0.75)	**<0.001**	0.71 (0.46–1.09)	0.119
Low Dose RT + LTADT vs. Low Dose RT	1.38 (1.02–1.87)	0.035	0.34 (0.22–0.54)	**<0.001**	0.35 (0.20–0.63)	**<0.001**
High Dose RT vs. Low Dose RT	1.24 (0.98–1.57)	0.074	0.86 (0.58–1.29)	0.475	0.68 (0.38–1.24)	0.209
High Dose RT + STADT vs. Low Dose RT	1.47 (0.95–2.26)	0.083	0.63 (0.20–1.96)	0.427	N/A	N/A
*Comparisons with Low Dose RT* *+* *STADT*						
Low Dose RT + LTADT vs. Low Dose RT + STADT	1.24 (0.96–1.61)	0.095	0.62 (0.42–0.91)	0.016	0.50 (0.30–0.81)	**0.005**
High Dose RT vs. Low Dose RT + STADT	1.12 (0.89–1.40)	0.336	1.56 (1.08–2.27)	0.019	0.96 (0.57–1.64)	0.89
High Dose RT + STADT vs. Low Dose RT + STADT	1.32 (0.88–1.99)	0.186	1.14 (0.39–3.38)	0.809	N/A	N/A
*Comparisons with High Dose RT*						
Low Dose RT + LTADT vs. High Dose RT	1.11 (0.82–1.51)	0.484	0.40 (0.25–0.64)	**<0.001**	0.52 (0.27–0.99)	0.049
High Dose RT + STADT vs. High Dose RT	1.18 (0.79–1.77)	0.424	0.73 (0.24–2.19)	0.575	N/A	N/A

*CI* confidence interval, *DM* distant metastasis, *LTADT* long-term ADT, *OR* odds ratio, *OS* overall survival, *PCSM* prostate cancer-specific mortality, *RT* radiation therapy, *STADT* short-term ADT.

Results are adjusted for median age, percentage of high-risk patients, and year of study using the midpoint of study enrollment. *P*-value thresholds for significance were 0.006 for OS and PCSM, and 0.008 for DM.

Meta-regression analyses were performed using the DerSimonian–Laird random-effects model to evaluate 5-year cumulative proportions for OS, PCSM, and DM across different treatment strategies [5]. Inter-study heterogeneity was evaluated using an *I*^2^ measure after accounting for covariates. The proportion of the total inter-study variability that was explained by covariates was quantified using the *R*^2^ measure. A Bonferroni correction was added to account for multiple testing within a given outcome. The null hypothesis was rejected at *P* < 0.006 for OS and PCSM (9 pairwise comparisons) or *P* < 0.008 level for DM (6 pairwise comparisons) thus controlling the type 1 error rate at <5%. All analyses were performed using the Metafor package in R version 4.0.2.

Forty trials recruiting a total of 21,429 patients met our inclusion criteria after reviewing by two investigators (TJ and AUK) (Supplementary Tables 1 and 2). The median follow-up for all trial arms was 9.15 years. The results of the meta-regression evaluating the impact of the time period, age, and percentages of patients with the high-risk disease on each outcome, stratified by treatment strategy, are shown in Supplementary Table 3. Covariate adjusted 5-year OS, PCSM, and DM proportions are shown in Supplementary Fig. 2, and results of the corresponding meta-regression comparing 5-year cumulative proportions using the log-odds scale across different strategies are shown in Table 1. No significant differences in OS were observed for any comparison. There was no significant difference in PCSM (odds ratio [OR] 0.86, 95% confidence interval [CI] 0.58–1.29), or DM (OR 0.68, 95% CI 0.38–1.24] when comparing high dose RT alone vs low dose RT alone.

Adding either STADT or LTADT to low dose RT alone improved 5-year PCSM (OR 0.55, 95% CI 0.41–0.75 and 0.34, 95% CI 0.22–0.54, respectively) and DM for LTADT (OR 0.35, 95% CI 0.20–0.63). When considering low dose RT + STADT as the reference group, prolongation to LTADT improved 5-year DM (OR 0.50, 95% CI 0.30–0.81). Low dose RT + STADT did not differ in any of the reported outcomes when compared to high dose RT alone or high dose RT + STADT. Adding STADT to high dose RT was not associated with a significant change in OS or PCSM (DM outcomes not evaluable due to fewer studies explicitly reporting proportions). When looking at 10-year outcomes, low dose RT + STADT no longer showed statistical DM and PCSM benefit when compared to low dose RT alone and high dose RT alone respectively. However, low dose RT + LTADT showed statistical improvement in 10-year PCSM when compared to low dose RT + STADT (Supplementary Table 4). No statistical differences were observed when using 76 Gy as the threshold for high dose RT for 5-year outcomes (Supplementary Table 5).

The results of this meta-analysis suggest that the benefit of adding STADT and LTADT to low-dose RT outweighs the benefit of solely intensifying the dose of radiation. Escalating RT dose in the absence of ADT did not demonstrate any significant improvement in OS, PCSM, or DM, and appeared to be associated with worse outcomes compared to low dose RT + LTADT. On the other hand, high dose RT was not significantly different from low dose RT + STADT for any outcomes. While the present analysis did not identify any significant benefit to adding STADT to high dose RT, this analysis is likely underpowered to detect any such difference given the comparatively few trials eligible for analysis, and as of now data from the TROG RADAR trial, EORTC 22991, and PCS III do no support this observation. Additionally, both forms of treatment intensification are associated with an increased risk of toxicity and should be used when clinically beneficial outcomes are expected [6]. Notably, as a radiosensitizer, ADT increases local control, but can also have cytostatic, if not cytocidal, effects on micrometastatic disease [7]. Dose escalation improves local control [1] and might thus abrogate a later “wave” of distant metastases, but would not address occult micrometastatic disease at presentation [8].

A major limitation of this meta-analysis is the lack of individual patient data. We could not account for all the heterogeneity in baseline characteristics between or within studies, and the lack of individual patient data precluded the inclusion of some key studies. Between-study heterogeneity, as measured by *I*^2^, was high for most treatment arm estimates for PCSM and DM, suggesting significant differences in study-level covariates that we were unable to account for. The small number of trials utilizing high dose RT + STADT limit the ability to detect differences between this treatment and others.

Overall, these data suggest the benefits offered by ADT to low dose RT exceed that of increasing radiation dose alone. Dose-escalation to greater than 74 Gy in the presence of STADT also does not appear to meaningfully improve outcomes, though there was also no clear evidence that omitting STADT in the presence of high dose RT worsened outcomes. Individual patient data meta-analyses of existing trials, and further studies of novel biomarkers in new trials, will help clarify the relative benefit of dose escalation in the context of STADT.

## Supplementary information

Supplementary Table and Figure Legends

Supplemental Files

Supplemental Figure 2
