# Supplementary material for: Radiation therapy dose and androgen deprivation therapy in localized prostate cancer: a meta-regression of 5-year outcomes in phase III randomized controlled trials
Source: Prostate Cancer Prostatic Dis. 2021 Aug 16;25(1):126–8. doi: 10.1038/s41391-021-00432-2 (PMC9018418; doi:10.1038/s41391-021-00432-2)
Supplement: Supplementary file 1 — Supplementary Table and Figure Legends [file 41391_2021_432_MOESM1_ESM.docx]

**Supplementary Figure 1:** Preferred Reporting Items for Systematic Reviews and Meta-Analyses Flowchart

**Supplementary Figure 2**: Adjusted 5-year cumulative proportions of overall survival, prostate cancer-specific mortality, and distant metastases for the various treatment strategies. Adjustments were made for median age, percentage of patients with high risk prostate cancer, and year of study (by using midpoint of study enrollment).

**Supplementary Table 1:** Summary of Trials Included in Study (By Arm)

**Supplementary Table 2:** Excluded Trials/Arms

**Supplementary Table 3:** Meta-regression for evaluating 5-Year Overall Survival, 5-Year Prostate Cancer Specific Mortality, and 5-Year Distant Metastasis across different radiation therapy-based treatments.

**Supplementary Table 4:** Adjusted meta-regression comparing 10-year cumulative proportions of Overall Survival, Prostate Cancer Specific Mortality, and Distant Metastasis.

**Supplementary Table 5:** Adjusted meta-regression comparing 5-year cumulative proportions of Overall Survival, Prostate Cancer Specific Mortality, and Distant Metastasis using *76 Gy as the threshold for high dose radiation therapy*.
