# Supplementary material for: Radiation therapy dose and androgen deprivation therapy in localized prostate cancer: a meta-regression of 5-year outcomes in phase III randomized controlled trials
Source: Prostate Cancer Prostatic Dis. 2021 Aug 16;25(1):126–8. doi: 10.1038/s41391-021-00432-2 (PMC9018418; doi:10.1038/s41391-021-00432-2)
Supplement: Supplementary file 2 — Supplemental Files [file 41391_2021_432_MOESM2_ESM.docx]

**Supplementary Figure 1:** Preferred Reporting Items for Systematic Reviews and Meta-Analyses Flowchart

## Identification

## Eligibility

## Included

## Screening

Records identified through database searching
(n = 1488)

Additional records identified through other sources
(n = 26)

Records after duplicates removed
(n = 1148)

Records screened
(n = 1148)

Records excluded
(n = 1092)

Full-text articles assessed for eligibility
(n = 56)

Studies included in qualitative synthesis
(n = 40)

Studies included in quantitative synthesis (meta-analysis)
(n = 40)

Full-text articles excluded, with reasons
(n = 19)

Variable Proportions of ADT: 8

Lifelong ADT: 2

ADT Not Categorizable: 2

Non-uniform dosage: 2

Missing outcomes: 2

**Supplementary Table 1:** Summary of Trials Included in Study (By Arm)

| Trial Name | PMID | Radiation Dose (Gy) | ADT Duration (Months) | Patients | Age | Midpoint of Enrollment | Percent High Risk | Follow-up  (Median, years) |
| --- | --- | --- | --- | --- | --- | --- | --- | --- |
| **Low Dose RT Alone** | | | | | | | | |
| RTOG 8531 | 15817329 | 65 | N/A | 489 | 70 | September 1989 | 100% | 11 |
| RTOG 8610 | 18172188 | 65-70 | N/A | 232 | 71 | May 1989 | 100% | 13.2 |
| RT0G 9408 | 21751904 | 66.6 | N/A | 992 | 71 | December 1997 | 11% | 9.2 |
| RTOG 0126 | 29543933 | 70.2 | N/A | 751 | 71 | May 2005 | 0% | 8.8 |
| RTOG 0415 | 27044935 | 70 | N/A | 550 | 67 | January 2008 | 0% | 5.8 |
| GETUG 06 | 21147514 | 70 | N/A | 153 | 67 | November 2000 | 28% | 5.1 |
| PROG 95-09 | 16160131+  20124169 | 70.2 | N/A | 197 | 67 | December 1997 | 5.1% | 8.9 |
| Lukka | 16135479 | 66 | N/A | 470 | 70.3 | January 1997 | 0% | 5.7 |
| Lukka | 16135479 | 52.5 | N/A | 466 | 70 | January 1997 | 0% | 5.7 |
| Adelaide | 16965866 | 55 | N/A | 108 | 69 | January 2000 | N/A | 4 |
| Adelaide | 16965866 | 64 | N/A | 109 | 69 | January 2000 | N/A | 4 |
| MDACC Dose Escalation | 12128107+  17765406 | 70 | N/A | 150 | 69 | June 1995 | 32% | 9.5 |
| TROG 9601 | 16257791+  21440505 | 66 | N/A | 270 | 67 | April 1998 | 82% | 10.6 |
| D’Amico | 18212313+  26393854 | 70.35 | N/A | 104 | 73 | August 1998 | 27.9% | 16.62 |
| EORTC 22863 | 12126818+  20933466 | 70 | N/A | 208 | 70 | July 1991 | 100% | 9.1 |
| MGH Proton Boost Trial | 7721636 | 67.2 | N/A | 99 | 68.6 | January 1987 | 95% | 5.1 |
| Ontario | 15718316+  28816169 | 66 | N/A | 53 | 66 | February 1995 | 60% | 14 |
| **High Dose Radiation Alone** | | | | | | | | |
| RTOG 0126 | 29543933 | 79.2 | N/A | 748 | 71 | May 2005 | 0% | 8.8 |
| GETUG 06 | 21147514 | 80 | N/A | 153 | 67 | November 2000 | 29% | 5.1 |
| PROG 95-09 | 16160131+  20124169 | 79.2 | N/A | 195 | 66 | December 1997 | 4% | 8.9 |
| PROFIT | 28296582 | 60 | N/A | 608 | 72 | January 2008 | 0% | 6 |
| PROFIT | 28296582 | 78 | N/A | 598 | 71 | January 2008 | 0% | 6 |
| RTOG 0415 | 27044935 | 73.8 | N/A | 542 | 67 | January 2008 | 0% | 5.8 |
| EORTC 22991 | 26976418 | 70-78 | N/A | 409 | 70 | December 2004 | 37.4% | 7.2 |
| MDACC Dose Escalation | 12128107+  17765406 | 78 | N/A | 151 | 69 | June 2005 | 35% | 9.5 |
| MGH Proton Boost Trial | 7721636 | 75.6 | N/A | 103 | 70 | January 1987 | 95% | 5.1 |
| HYPO-RT-PC | 31227373 | 42.7 | N/A | 589 | 68 | August 2010 | 11% | 5 |
| HYPO-RT-PC | 31227373 | 78 | N/A | 591 | 69 | August 2010 | 11% | 5 |
| PMH 9907 | 27219522 | >75.6 | N/A | 122 | 70.9 | July 2002 | 6.5% | 9.1 |
| PCS III | 33279855 | 76 | N/A | 200 | 71 | October 2005 | N/A | 11.3 |
| **Low Dose with Short-Term ADT** | | | | | | | | |
| RTOG 8610 | 18172188 | 65-70 | 4 | 224 | 70 | May 1989 | 100% | 11.9 |
| RTOG 9202 | 14581419+  18413638 | 65-70 | 4 | 763 | 70 | October 1993 | 100% | 11.31 |
| RTOG 9408 | 21751904 | 66.6 | 4 | 987 | 70 | December 1997 | 11% | 9.1 |
| RTOG 9413 | 12743142 | 70 | NHT+WPRT | 318 | 71 | May 1997 | 79.7% | 13.93 |
| RTOG 9413 | 12743142 | 70 | NHT+PORT | 316 | 71 | May 1997 | 79.7% | 14.26 |
| RTOG 9413 | 12743142 | 70 | WPRT+AHT | 319 | 71 | May 1997 | 79.7% | 15.56 |
| RTOG 9413 | 12743142 | 70 | PORT+AHT | 317 | 71 | May 1997 | 79.9% | 15.77 |
| RTOG 9910 | 25534388 | 70.2 | 4 | 752 | 71 | March 2002 | 15% | 9.4 |
| RTOG 9910 | 25534388 | 70.2 | 9 | 737 | 71 | March 2002 | 15% | 9.4 |
| MRC RT 101 | 17482880+  24581940 | 64 | 3-6 | 421 | 67 | June 2000 | 43% | 5.3 |
| TROG 9601 | 16257791+  21440505 | 66 | 6 | 267 | 68 | April 1998 | 85% | 10.6 |
| D’Amico | 18212313+  26393854 | 70. 35 | 6 | 102 | 72.5 | August 1998 | 25.5% | 16.62 |
| EORTC 22961 | 19516032 | 70 | 6 | 483 | 70 | July 1999 | 74.6% | 6.4 |
| ICORG 97-01 | 20797824 | 70 | 4 | 137 | 67 | July 1999 | 47% | 8.5 |
| ICORG 97-01 | 20797824 | 70 | 8 | 139 | 67 | July 1999 | 46% | 8.5 |
| PCS III | 33279855 | 70 |  | 200 | 71 | October 2005 | N/A | 11.3 |
| Low Dose with Long-Term ADT | | | | | | | | |
| RTOG 9202 | 14581419+  18413638 | 65-70 | 24 | 758 | 70 | October 1993 | 100% | 11.31 |
| RTOG 9902 | 26209502 | 70.2 | 24 | 197 | 65 | May 2002 | 100% | 10 |
| RTOG 9902 | 26209502 | 70.2 | 24 | 200 | 66.5 | May 2002 | 100% | 10 |
| RTOG 0521 | 30860948 | 72-75.6 | 24 | 281 | 66 | October 2007 | 100% | 6.1 |
| PCS IV | 29980331 | 69.2-76 | 36 | 310 | 71 | May 2004 | 100% | 9.36 |
| PCS IV | 29980331 | 68-76 | 18 | 320 | 71 | May 2004 | 100% | 9.39 |
| EORTC 22863 | 12126818+  20933466 | 70 | 36 | 207 | 70 | July 1991 | 100% | 9.1 |
| EORTC 22961 | 19516032 | 70 | 36 | 487 | 69 | July 1999 | 71.5% | 6.4 |
| High Dose with Short-Term ADT | | | | | | | | |
| Malone | 31829912 | 76 | 6 | 215 | 71 | May 2007 | 3.7% | 12.35 |
| Malone | 31829912 | 76 | 6 | 217 | 69 | May 2007 | 5.5% | 11.93 |
| GICOR DART | 25702876 | >76 | 4 | 178 | 72 | May 2008 | 55% | 5.1 |
| PCS III | 33279855 | 76 | 6 | 200 | 71 | October 2005 | N/A | 11.3 |
| PROTECT | 27626136 | 74 | 3-6 | 545 | 62 | May 2005 | N/A | 10 |
| MRC RT 101 | 17482880+  24581940 | 74 | 3-6 | 422 | 67 | June 2000 | 44% | 10 |

**Supplementary Table 2:** Excluded Trials/Arms

| Trial or Trial Arm | PMID | Reason |
| --- | --- | --- |
| GICOR DART | 25702876 | Only trial with uniform high dose RT + LTADT |
| TROG 9601 RT+3-month ADT | 21440505 | 3 months duration had mixed effect (improved biochemical recurrence but not DM or PCSM) |
| TROG RADAR | 30579763 | Non-uniform dose in both arms |
| EORTC 22991 | 26976418 | Non-uniform dose in both arms |
| ASCENDE RT | 28262473 | Non-standard ADT duration (12 months) in both arms, as well as use of brachytherapy boost in one arm |
| Regina Elena | 28355113 | Non-standard ADT duration (9 months) in both arms |
| Ontario Arm 2 EBRT+BT | 15718316+28816169 | This arm used a brachytherapy boost |
| Mt. Vernon | 33011207 | Non-uniform ADT usage in both arms, as well as use of brachytherapy boost in one arm |
| PMH 9907 RT+Bicalutamide arm | 27219522 | Non-standard ADT agent (bicalutamide monotherapy) |
| MDACC Hypofractionation Trial | 30106637 | Non-uniform ADT usage in both arms |
| CHHiP | 27339115 | Non-uniform ADT usage in both arms, as well as bicalutamide monotherapy in some patients receiving ADT (non-standard ADT agent) |
| Dutch CKTO 69-10 | 16648499 | Non-uniform ADT usage in both arms |
| HYPRO | 27339116 | Non-uniform ADT usage in both arms |
| FCCC | 24101042 | Non-uniform ADT usage in both arms |
| GETUG 01 | 27788949 | Non-uniform ADT usage in both arms |
| Quebec L-101 | [14767287](https://www.ncbi.nlm.nih.gov/pubmed/14767287?dopt=Abstract) | Does not report many outcomes |
| Quebec L-200 | - 14767287 | Does not report many outcomes |
| Canada Multicenter | 18707821 | Does not report many outcomes |
| RTOG 8531 Arm 2 (RT+lifelong) | 15817329 | Non-standard AT duration (lifelong) |
| SPCG-7 Arm 2 (RT+lifelong) | 27025586 | Non-standard AT duration (lifelong) |
| NCIC Warde Arm 2 (RT+lifelong) | 25691677 | Non-standard AT duration (lifelong) |

**Supplementary Table 3:** Meta-regression for evaluating 5-Year Overall Survival, 5-Year Prostate Cancer Specific Mortality, and 5-Year Distant Metastasis across different radiation therapy-based treatments.

|  | 5-yr OS | | 5-yr PCSM | | 5-yr DM | |
| --- | --- | --- | --- | --- | --- | --- |
|  | **OR (95% CI)** | p-value | **OR (95% CI)** | p-value | **OR (95% CI)** | p-value |
| *Treatment: Low Dose Radiation Therapy* | | | | | | |
| Middle of Study Enrollment | 1.01 (0.97-1.05) | 0.582 | 0.93 (0.86-1.01) | 0.066 | 1.05 (0.98-1.12) | 0.156 |
| Median Age of Patient | 0.81 (0.75-0.88) | **< 0.001** | 1.11 (0.95-1.29) | 0.205 | 1.14 (1.00-1.30) | 0.053 |
| Percentage of High-Risk Patients | 0.99 (0.98-0.99) | **< 0.001** | 1.02 (1.01-1.03) | **< 0.001** | 1.04 (1.03-1.04) | **< 0.001** |
|  | I^2^ 62.3 | | I^2^ 82.9 | | I^2^ 96.2 | |
|  | R^2^ 90.0 | | R^2^ 76.9 | | R^2^ 38.5 | |
| *Treatment: High Dose Radiation Therapy* | | | | | | |
| Middle of Study Enrollment | 1.03 (0.99-1.08) | 0.172 | 0.91 (0.86-0.96) | **< 0.001** | 0.88 (0.75-1.03) | 0.105 |
| Median Age of Patient | 0.84 (0.76-0.92) | **< 0.001** | N/A | N/A | N/A | N/A |
| Percentage of High-Risk Patients | 0.99 (0.98-1.01) | 0.298 | N/A | N/A | N/A | N/A |
|  | I^2^ 60.0 | | I^2^ 84.6 | | I^2^ 85.0 | |
|  | R^2^ 74.4 | | R^2^ 15.0 | | R^2^ 34.0 | |
| *Treatment: Low Dose Radiation Therapy with Short-Term Androgen Deprivation Therapy* | | | | | | |
| Middle of Study Enrollment | 1.03 (1.00-1.07) | 0.077 | 0.95 (0.89-1.02) | 0.171 | 0.90 (0.81-0.99) | **0.036** |
| Median Age of Patient | 0.92 (0.87-0.98) | **0.009** | 0.93 (0.81-1.07) | 0.303 | 0.87 (0.75-1.02) | 0.079 |
| Percentage of High-Risk Patients | 0.99 (0.99-1.00) | **0.001** | 1.02 (1.01-1.03) | **< 0.001** | 1.01 (1.00-1.02) | 0.131 |
|  | I^2^ 19.3 | | I^2^ 39.3 | | I^2^ 72.1 | |
|  | R^2^ 95.4 | | R^2^ 93.7 | | R^2^ 87.1 | |
| *Treatment: Low Dose Radiation Therapy with Long-Term Androgen Deprivation Therapy* | | | | | | |
| Middle of Study Enrollment | 1.04 (1.02-1.07) | **0.001** | 0.99 (0.92-1.06) | 0.72 | 0.99 (0.95-1.05) | 0.805 |
| Median Age of Patient | N/A | N/A | N/A | N/A | N/A | N/A |
| Percentage of High-Risk Patients | N/A | N/A | N/A | N/A | N/A | N/A |
|  | I^2^ 41.6 | | I^2^ 75.2 | | I^2^ 72.5 | |
|  | R^2^ 77.0 | | R^2^ 0 | | R^2^ 0 | |

CI, confidence interval; DM, distant metastasis; LTADT, long-term ADT; OR, odds ratio; OS, overall survival; PCSM, prostate cancer-specific mortality; RT, radiation therapy; STADT, short-term ADT

Odds ratios correspond to 1-year increase in midpoint of study enrollment, 1- year increase in median age of study participants, and 1 percentage point increase in patients diagnosed with high-risk disease.

**Supplementary Table 4:** Adjusted meta-regression comparing 10-year cumulative proportions of Overall Survival, Prostate Cancer Specific Mortality, and Distant Metastasis.

|  | 10-yr OS | | 10-yr PCSM | | 10-yr DM | |
| --- | --- | --- | --- | --- | --- | --- |
|  | **OR (95% CI)** | p-value | **OR (95% CI)** | p-value | **OR (95% CI)** | p-value |
| *Comparisons with Low Dose RT* | | | | | | |
| Low Dose RT + STADT vs. Low Dose RT | 1.14 (0.89-1.46) | 0.289 | 0.58 (0.43-0.78) | **< 0.001** | 0.74 (0.48-1.15) | 0.177 |
| Low Dose RT + LTADT vs. Low Dose RT | 1.45 (0.98-2.15) | 0.066 | 0.29 (0.18-0.46) | **< 0.001** | 0.49 (0.27-0.88) | 0.017 |
| High Dose RT vs.  Low Dose RT | 1.61 (1.06-2.45) | 0.025 | N/A | N/A | N/A | N/A |
| High Dose RT + STADT vs. Low Dose RT | 1.48 (0.96-2.28) | 0.077 | 0.77 (0.44-1.32) | 0.339 | 0.89 (0.42-1.87) | 0.759 |
| *Comparisons with Low Dose RT + STADT* | | | | | | |
| Low Dose RT + LTADT vs. Low Dose RT + STADT | 1.27 (0.92-1.75) | 0.151 | 0.50 (0.34-0.73) | **< 0.001** | 0.66 (0.42-1.06) | 0.085 |
| High Dose RT vs.  Low Dose RT + STADT | 1.41 (0.93-2.13) | 0.103 | N/A | N/A | N/A | N/A |
| High Dose RT + STADT vs. Low Dose RT + STADT | 1.29 (0.89-1.89) | 0.183 | 1.33 (0.81-2.18) | 0.260 | 1.21 (0.64-2.27) | 0.563 |
| *Comparisons with High Dose RT* | | | | | | |
| Low Dose RT + LTADT vs. High Dose RT | 0.90 (0.52-1.55) | 0.699 | N/A | N/A | N/A | N/A |
| High Dose RT + STADT vs. High Dose RT | 0.92 (0.55-1.52) | 0.737 | N/A | N/A | N/A | N/A |

CI, confidence interval; DM, distant metastasis; LTADT, long-term ADT; OR, odds ratio; OS, overall survival; PCSM, prostate cancer-specific mortality; RT, radiation therapy; STADT, short-term ADT

Results are adjusted for median age, percentage of high-risk patients, and year of study using the midpoint of study enrollment. P-value thresholds for significance were 0.006 for OS and PCSM, and 0.008 for DM.

**Supplementary Table 5:** Adjusted meta-regression comparing 5-year cumulative proportions of Overall Survival, Prostate Cancer Specific Mortality, and Distant Metastasis using *76 Gy as the threshold for high dose radiation therapy*.

|  | 5-yr OS | | 5-yr PCSM | | 5-yr DM | |
| --- | --- | --- | --- | --- | --- | --- |
|  | **OR (95% CI)** | p-value | **OR (95% CI)** | p-value | **OR (95% CI)** | p-value |
| *Comparisons with Low Dose RT* | | | | | | |
| Low Dose RT + STADT vs. Low Dose RT | 1.06 (0.89-1.28) | 0.517 | 0.59 (0.44-0.78) | **< 0.001** | 0.71 (0.50-1.01) | 0.054 |
| Low Dose RT + LTADT vs. Low Dose RT | 1.31 (0.98-1.75) | 0.068 | 0.36 (0.23-0.56) | **< 0.001** | 0.35 (0.21-0.57) | **< 0.001** |
| High Dose RT vs.  Low Dose RT | 1.20 (0.93-1.55) | 0.172 | 0.93 (0.58-1.50) | 0.777 | 0.51 (0.30-0.88) | 0.015 |
| High Dose RT + STADT vs. Low Dose RT | 1.39 (0.91-2.12) | 0.132 | N/A | N/A | N/A | N/A |
| *Comparisons with Low Dose RT + STADT* | | | | | | |
| Low Dose RT + LTADT vs. Low Dose RT + STADT | 1.23 (0.96-1.59) | 0.108 | 0.62 (0.43-0.90) | 0.013 | 0.49 (0.32-0.76) | **< 0.001** |
| High Dose RT vs.  Low Dose RT + STADT | 1.13 (0.87-1.46) | 0.365 | 1.60 (1.02-2.48) | 0.041 | 0.71 (0.42-1.20) | 0.207 |
| High Dose RT + STADT vs. Low Dose RT + STADT | 1.30 (0.86-1.97) | 0.206 | N/A | N/A | N/A | N/A |
| *Comparisons with High Dose RT* | | | | | | |
| Low Dose RT + LTADT vs. High Dose RT | 1.09 (0.79-1.53) | 0.596 | 0.39 (0.23-0.66) | **< 0.001** | 0.69 (0.36-1.31) | 0.256 |
| High Dose RT + STADT vs. High Dose RT | 1.16 (0.76-1.76) | 0.495 | N/A | N/A | N/A | N/A |

CI, confidence interval; DM, distant metastasis; LTADT, long-term ADT; OR, odds ratio; OS, overall survival; PCSM, prostate cancer-specific mortality; RT, radiation therapy; STADT, short-term ADT

Results are adjusted for median age, percentage of high-risk patients, and year of study using the midpoint of study enrollment. P-value thresholds for significance were 0.006 for OS and PCSM, and 0.008 for DM.
