# Supplementary figures and images for: Radiation therapy dose and androgen deprivation therapy in localized prostate cancer: a meta-regression of 5-year outcomes in phase III randomized controlled trials
Source: Prostate Cancer Prostatic Dis. 2021 Aug 16;25(1):126–8. doi: 10.1038/s41391-021-00432-2 (PMC9018418; doi:10.1038/s41391-021-00432-2)

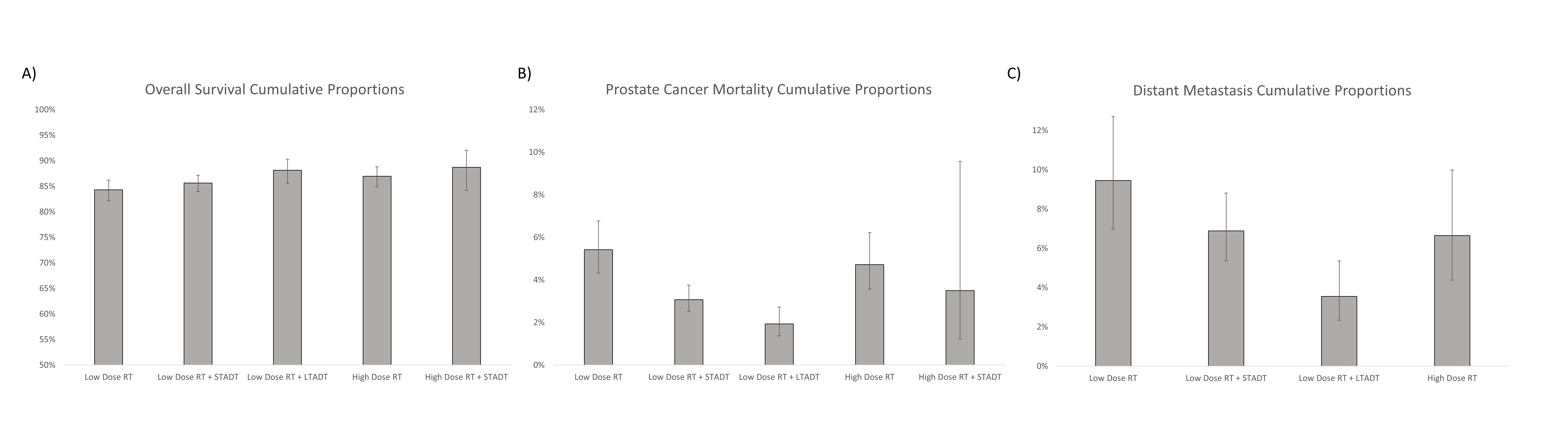

Supplement: Supplementary file 3 — Supplemental Figure 2 [file 41391_2021_432_MOESM3_ESM.tif]
